# Limb Salvage in Chondrosarcoma of the Proximal Humerus: A Case Report

**DOI:** 10.7759/cureus.64266

**Published:** 2024-07-10

**Authors:** Devishi Sarin, Kuldeep Bansal, Anuj Gupta

**Affiliations:** 1 Orthopedics, Fudan University, Shanghai, CHN; 2 Orthopedics, University College of Medical Sciences, Delhi, IND; 3 Orthopedics and Spine, Triveni Ortho and Spine Center, Delhi, IND; 4 Spine Surgery, Max Super Speciality Hospital, Delhi, IND

**Keywords:** autoclave, malignancy surgery, bone tumors, proximal humerus, primary chondrosarcoma

## Abstract

Chondrosarcoma is the third most common primary malignant bone tumor. The proximal humerus is the most common site. Since it is resistant to chemotherapy and radiotherapy, the mainstay of treatment is surgery. Due to the extensive involvement of long bones, it requires reconstruction with either a prosthetic implant or bone graft. We present a case of a 43-year-old female who presented with chondrosarcoma involving 15 cm of humerus. The patient was managed with the resection of 15 cm of humerus and reconstruction with the same resected bone after autoclaving. It was secured with long fixation resulting in arthrodesis of the glenohumeral joint. The patient was followed for one year and there was evidence of callus formation by ultrasound and computed tomography (CT) scan.

## Introduction

Chondrosarcoma is the third most common primary malignant tumor of the bone after myeloma and osteosarcoma. It accounts for 20% of all sarcomas [1]. The humerus is the most common site for chondrosarcoma [2]. However, this tumor is known for its resistance to radiotherapy and chemotherapy [3]. Therefore, surgery is the best and only option available for its management.

Chondrosarcoma is a malignant tumor and, therefore, can progress rapidly if not treated in the early stages. The surgical management requires removal with the care that the resected part of the bone contains tumor-free margins [4]. However, the location and size of the tumor are the most important factors for the outcome. Most chondrosarcoma involves the proximal part of the humerus. The tumor resection may require the removal of soft tissue structures like the rotator cuff, deltoid, and other ligaments [5]. After that, reconstruction is needed to improve the quality of life of the patient. There are various methods of reconstruction, especially if the tumor size is large: allograft [5], irradiated autograft [6], and autoclaved autograft [7]. Apart from these, prostheses for reconstruction are available too.

In developing countries, a lot of patients are not able to afford tumor prostheses, and allografts are not easily available. We present a case of chondrosarcoma involving a large part of the humerus, treated with resection of the tumor and reconstruction with an autoclaved autograft.

## Case presentation

**Figure 1:**
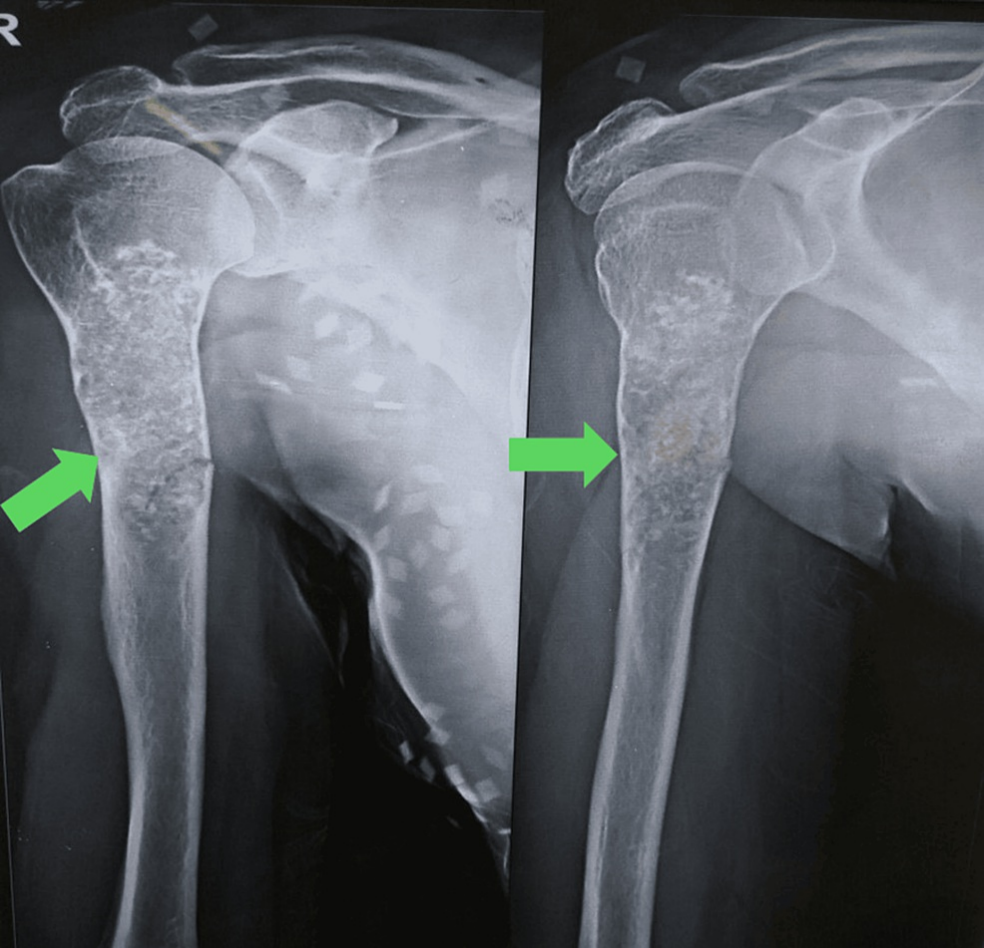
Pre-operative radiographs showing chondrosarcoma involving part of the humerus

Our case is a 43-year-old female presented with biopsy-proven chondrosarcoma involving 15 cm of humerus including the proximal part. Her main complaint was pain in her arm and restricted shoulder range of motion due to pain. There was no distal neurovascular deficit. Radiographs were performed to look for the extent of the tumor and the involvement of neurovascular bundles. The radiographs showed 15 cm of involvement of the proximal humerus by tumor (Figure 1).

**Figure 2:**
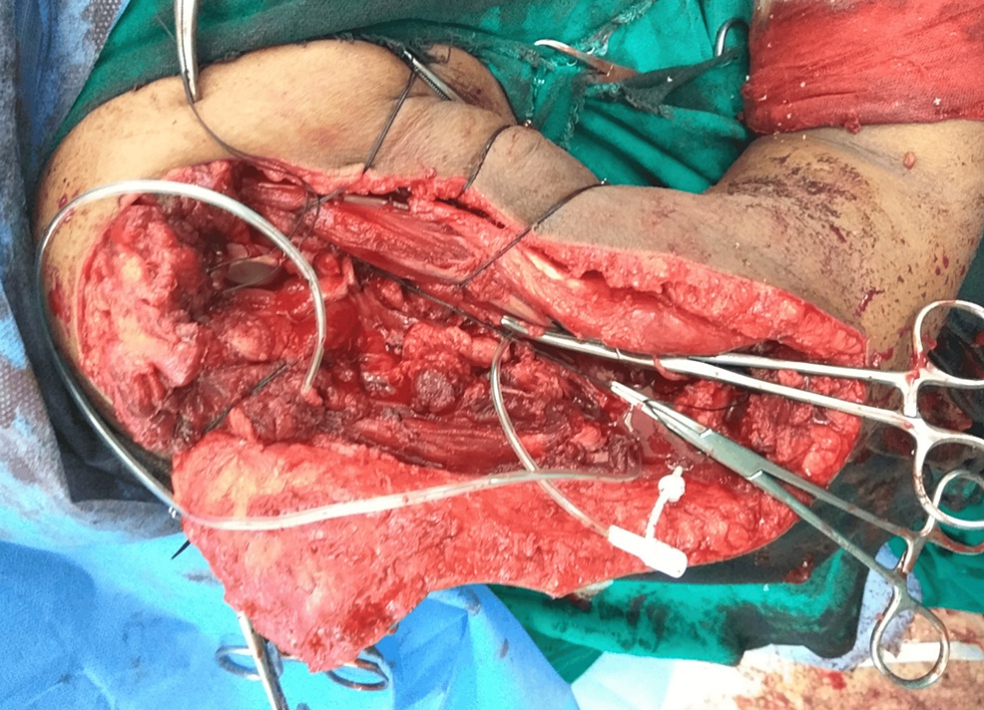
Intra-operative picture after resection of the bone

The surgery was planned and the patient was explained about the procedure and arthrodesis. Enbloc resection was performed and deltoid muscle was sacrificed as it was found involved in the tumor tissue (Figure 2).

**Figure 3:**
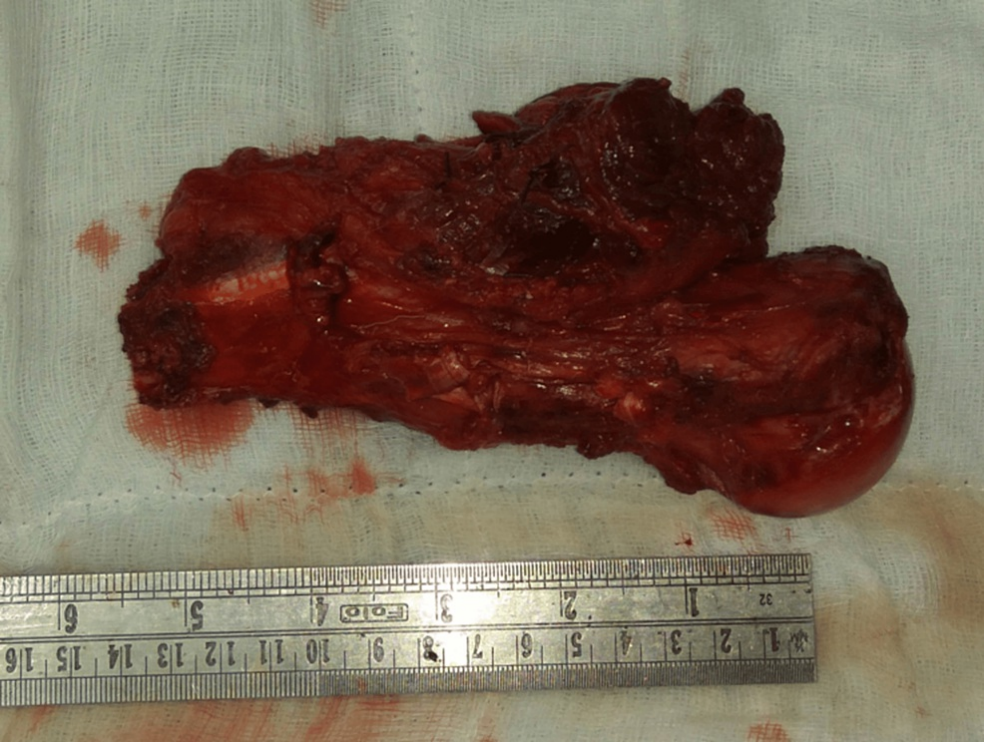
Resected part of the humerus

The resected bone with tumor was cleaned of soft tissue and autoclaved at 120°C for 10 mins (Figure 3).

**Figure 4:**
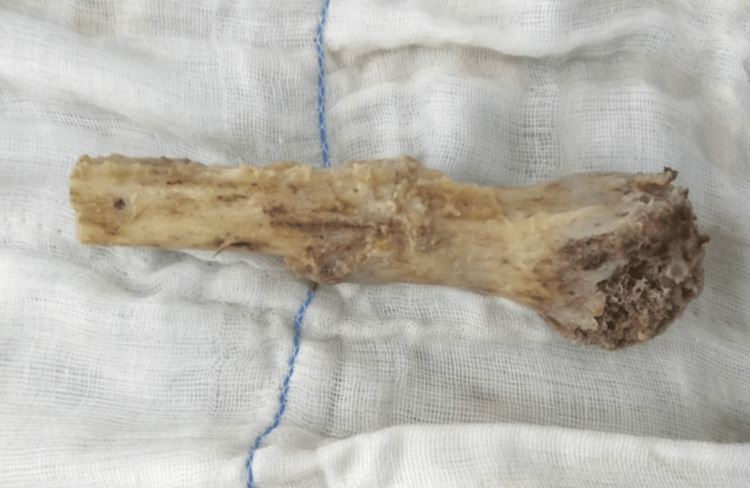
Resected part of the humerus after autoclaving

Then it was cleaned with copious saline and the tumor was curetted (Figure 4).

**Figure 5:**
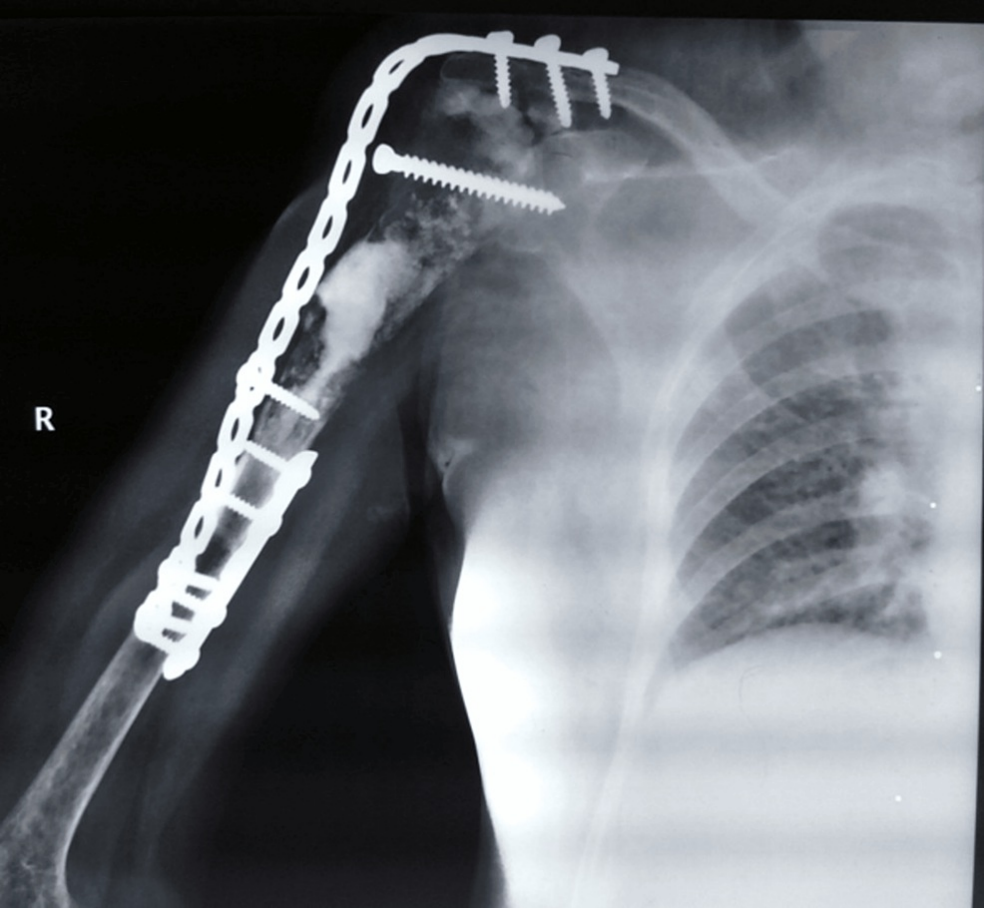
Radiographs at a one-year follow-up

The gap was filled with polymethylmethacrylate. It was reimplanted and shoulder arthrodesis was done using an 18-hole 4.5 mm recon plate from the spine of the scapula to the distal humerus. The junctional area was surrounded with prolene mesh with cortico-cancellous graft filled in it. The remnant of rotator cuff muscles was sutured with prolene mesh using ethibond. Post-operatively, the shoulder spica was applied and a window was cut for cleaning and dressing of the wound. Suture removal was done after two weeks and shoulder spica was continued. The patient was followed up for one year when this manuscript was written. The evaluation was done with serial radiographs (Figure 5).

In between computed tomography (CT) scan and ultrasound were done to look for callus formation at the junctional area and humero-glenoid area. We found a callus at both junctions after approximately three months. The shoulder spica was removed after six months.

## Discussion

Surgical management is the mainstay for treatment of chondrosarcoma. Since it is a highly malignant tumor, therefore, removal of the complete tumor with disease-free margins is mandatory for successful treatment. Failure to do so may lead to a recurrence of the tumor. The most common site is the proximal humerus. The literature supports resection of tumors along with reconstruction which can provide functional ability to the patient. The surgical reconstruction aims to restore the structural integrity and functional capacity of the humerus. The various options for reconstruction are endoprosthetic reconstruction, allograft, autograft, and composite reconstruction. Endoprosthetic reconstruction has become the choice of reconstruction for most of the tumors of bone [8]. They provide immediate structural support and immediate mobilization. Also, the patient can achieve near-normal functional capacity [9]. Allograft is another option that also provides good structural compatibility and biological integration over time [10]. However, the availability of allograft is a challenge. Not all centers are equipped with facilities to store allografts. Gomez et al. published a case report for the successful treatment of chondrosarcoma of the humerus using hemicortical allograft. They have an excellent follow-up of 56 months with good functional outcomes [5]. However, they also mention the problems associated with allograft. The biggest limitation is non-union which has been a common cause of hardware failure and revision surgery [11]. Even if, there is union, the sections of allograft weaken over time and there are high chances of fracture [12].

Another option for reconstruction is using the same resected bone obtained from the patient. This has been explored by many surgeons and can be done successfully. However, the tumor tissue needs to be eliminated from the autograft to prevent recurrence. The various options are irradiation or autoclaving of the autograft. Chen et al. [6] published a series of malignant bone tumors where resection and reconstruction using autograft were done. Out of 14 cases in their series, 3 were having chondrosarcoma. They suggested extracorporeal irradiation of bone at 300 Gy is useful for halting the growth of malignant cells. They observed no local recurrence after implantation of irradiated bone. Although they didn’t encounter any infection in their series, they mentioned that infection is the biggest risk in this irradiation method. They also mentioned it took them 50 minutes to transport and irradiate after which the bone was available to implant. However, not all centers have a facility for irradiation with this efficiency in terms of time.

Autoclaving is a good alternative for preventing the growth of tumor cells and preventing recurrence. The advantages of using autograft are biological compatibility, it preserves the original bone architecture and it is cost-effective [13]. Smith et al. [7] presented a case series of eight patients of chondrosarcoma managed with reimplantation of autograft after autoclaving. One of the patients in their series has a follow-up of 24 years. This patient had chondrosarcoma of the femur and was in great functional condition at 24 years of follow-up. The autoclaving protocol used by the authors was different from what we have used. They used a temperature of 135°C for 12-15 minutes. However, we did that at 120°C for 10 minutes. We have followed our patient for one year now which is less than the follow-up of Smith et al.; however, we could not find anything in the literature that mentions the end effects of the difference in autoclaving. We used this temperature and time as we assumed that using a higher temperature for a longer duration would make the bone completely non-viable.

One case in Smith et al. [7] series underwent a total hip replacement. It was observed that the entire autoclaved bone was live except few necrotic spicules. This was also confirmed with histopathological examination. Out of eight cases in their series, five involved the proximal humerus. The same procedure was done in all of them and on long-term follow-up, they have acceptable functional mobility at the glenohumeral joint. Due to extensive involvement by the tumor tissue in our patient, we found the glenohumeral joint was non-salvageable. Therefore, we decided to extend our fixation and fuse the joint. Another reason for the fusion of the glenohumeral joint was to get a mechanical advantage. Since, the resected bone was around 15 cm and autoclaving reduced the mechanical strength of the bone, therefore, long spanning by implant would give better mechanical strength to the whole construct. As a result, we could not comment on the functional outcome of the patient. However, we confirmed the presence of callus formation using ultrasound and CT scan at three months. This was similar to the outcome in the case series of Smith et al. [7] where the callus is visible in all cases at 2-3 months.

The main challenges with autoclaved bone are it loses its mechanical properties which increases the chances of fracture, it loses its biological properties as autoclaving also destroys living cells which can increase the chances of non-union and infection risk. Adjuvant therapies like bone grafting and the use of bone morphogenetic protein (BMP) can reduce the risk of non-union and can improve mechanical strength [14,15]. However, more long-term studies are required to look at the success of this procedure and to suggest modifications to make it better.

## Conclusions

Reconstruction with autoclaved bone is a viable option for limb salvage in patients with chondrosarcoma of the humerus. While it offers the advantages of biological compatibility and cost-effectiveness, it also presents challenges that need to be carefully managed. Advances in surgical techniques and post-operative care continue to improve the outcomes of this reconstruction method, making it a valuable tool in the armamentarium of orthopedic oncologists.
